# Assessing the Impact of Tropicamide on Anterior Segment Parameters in Diabetic Patients: A Randomized Clinical Trial

**DOI:** 10.7759/cureus.58223

**Published:** 2024-04-14

**Authors:** Navid Elmi Sadr, Seyyedeh Sedigheh Mirsharif, Joobin Khadamy, Samaneh Lavvaf, Ramyar Hariri

**Affiliations:** 1 Ophthalmology, Semnan University of Medical Sciences, Semnan, IRN; 2 Clinical Research Development Unit, Kowsar Educational, Research and Therapeutic Hospital, Semnan University of Medical Sciences, Semnan, IRN; 3 Ophthalmology, Skellefteå Eye Clinic, Skellefteå, SWE; 4 Ophthalmology, Norrlands Universitetssjukhus, Umeå, SWE; 5 Epidemiology, Semnan University of Medical Sciences, Semnan, IRN

**Keywords:** anterior chamber depth, contact lens fitting, refractive surgeries, intraocular surgeries, biometric measurements, tropicamide, pentacam, diabetes, cycloplegia, anterior segment

## Abstract

Introduction: Evaluation of anterior segment parameters is crucial in ophthalmic procedures such as intraocular surgeries and contact lens fitting. However, the use of tropicamide in diabetic patients presents challenges due to its potential impact on biometric measurements. This study aims to investigate and compare the effects of 0.5% and 1% tropicamide on anterior segment parameters in diabetic patients.

Methods: This double-masked randomized clinical trial enrolled 98 patients with diabetes mellitus. Participants were randomly assigned to receive either 0.5% or 1% tropicamide. Anterior segment parameters were measured using Pentacam HR (Oculus Optikgeräte GmbH, Wetzlar, Germany) before and 30 minutes after tropicamide administration. Parameters included anterior chamber depth (ACD), anterior chamber volume (ACV), anterior chamber angle (ACA), keratometry, central corneal thickness (CCT), white-to-white distance (WTW), and pupillary diameter (PD).

Results: Both concentrations of 0.5% and 1% tropicamide induced significant changes in anterior segment parameters. There was a notable increase in PD (2.99 ± 0.62, 3.11 ± 0.55, respectively, both P-values < 0.001), ACD (both 0.10 ± 0.05, both P-values < 0.001), ACV (16.69 ± 9.56, 17.51 ± 9.26, respectively, both P-values < 0.001), and WTW (0.06 ± 0.14, 0.03 ± 0.30, respectively, both P-values < 0.001), along with a decrease in ACA (-3.50 ± 10.65, -3.30 ± 6.87, P-value < 0.001 and P-value=0.001, respectively), and CCT (-6.10 ± 8.06, -6.39 ± 9.97, respectively, both P-values < 0.001) post-dilation. However, no significant changes were observed in keratometry (front Km (-0.03 ± 0.19, -0.04 ± 0.21, respectively), back Km (0.01 ± 0.05, 0.004 ± 0.05, respectively), P-values> 0.05).

Conclusion: Both concentrations of tropicamide exhibited comparable effects on anterior segment parameters in diabetic patients. These post-dilation changes should be considered for accurate intraocular lens power calculation and decision-making for cataract, phakic intraocular lens, and refractive surgeries.

## Introduction

In ophthalmic procedures, a detailed evaluation of the anterior segment holds immense importance, particularly in intraocular procedures, refractive surgeries, and contact lens fitting [1-3]. The advent of advanced optical coherence-based biometry devices has undoubtedly enhanced the precision of anterior segment assessments. Nevertheless, despite the widespread integration of these technologies, other technologies, such as the Pentacam HR rotating Scheimpflug camera (Oculus Optikgeräte GmbH, Wetzlar, Germany), alongside less advanced biometry devices, persist in certain clinical settings [4].

A fundamental step in assessing diabetic patients involves a mandatory fundus examination. However, time constraints in many clinics often lead to diabetic cases presenting themselves after self-administering pupil-dilating eye drops, such as tropicamide, or sometimes in the waiting room before consulting with a physician. This presents a significant challenge, particularly when patients are potential candidates for refractive surgeries or contact lens fitting, as tropicamide-induced pupil dilation can substantially influence biometric measurements. Consequently, many surgeons find it necessary to reschedule patients for separate visits to conduct accurate biometry assessments, adding to the challenges faced by busy clinical practices with limited resources.

While some studies have delved into the effects of cycloplegia on anterior segment parameters in healthy individuals [5], there remains a notable scarcity of research, particularly addressing this issue in diabetic patients. This gap is salient due to the unique challenges posed by diabetes [6,7], including structural and functional alterations in the anterior segment as well as diabetic autonomic neuropathy [8,9]. These factors suggest that diabetic eyes may respond differently to topical cycloplegia when compared to their healthy counterparts.

Accurate biometric measurements play a crucial role in guiding treatment decisions and reducing the incidence of postoperative refractive surprises or complications, particularly in diabetic patients. Understanding the influence of cycloplegia on anterior segment parameters is essential in this context. To assess any dose dependency of potential changes in anterior segment parameters, considering the reduced responsiveness to cycloplegics in diabetic patients, this study investigates the effects of two different commercially available doses of tropicamide. It aims to bridge this gap by evaluating and comparing the effects of either 0.5% or 1% tropicamide on anterior segment parameters using Pentacam HR.

## Materials and methods

Study design

This study adopts a double-masked randomized clinical trial design (ClinicalTrials.gov identifier (NCT number): NCT04932213) and was conducted at the Ophthalmology Clinic of Kowsar Hospital, affiliated with Semnan University of Medical Sciences, Semnan, Iran, from July 7, 2021, to November 7, 2021. The study adhered to the principles outlined in the Declaration of Helsinki and received approval from the Institutional Ethics Committee (IR.SEMUMS.REC.1400.018). Written informed consent was obtained from all participants. The dataset associated with this research may be found at: https://doi.org/10.7910/DVN/ODSLLZ

Participants

Patients aged 21 years and above with either type 1 or type 2 diabetes mellitus and any duration of diabetes referred from the Diabetes Clinic of Kowsar Hospital were screened for eligibility. Exclusion criteria included proliferative diabetic retinopathy, history of cataract surgery, severe nuclear and cortical cataracts, glaucoma, elevated intraocular pressure, narrow angles, pregnancy, pterygium, corneal ectasia, history of refractive surgery, corneal dystrophy, iris disorders, anisocoria, iris neovascularization, and use of miotics or mydriatics.

Study procedure

Participants were randomized into two parallel groups using the randomized block method, with one eye of each participant included in the study. Refraction and keratometry, visual acuity measurement, slit-lamp biomicroscopy, and Pentacam imaging were performed initially, followed by intraocular pressure measurement. In Group 1, patients received 0.5% tropicamide (one drop every five minutes for two doses), while in Group 2, patients received 1% tropicamide (one drop every five minutes for two doses). Thirty minutes later, all baseline examinations, along with dilated fundus examination, were repeated. A nurse administered the tropicamide drops, and both the participants and the ophthalmologist conducting the examinations were blinded to the concentration of tropicamide administered.

The Pentacam system utilizes a Scheimpflug camera to capture images while rotating 180 degrees, creating a three-dimensional image of the anterior segment used for parameter measurements. The software version utilized in this study was 1.22r09, and a single operator conducted all measurements. The automatic release mode was employed to ensure consistency and reliability, and only scans labeled as "OK" by the Pentacam's quality criteria were selected for analysis. Additionally, all examinations were performed between 10 a.m. and 1 p.m. to minimize potential variations due to diurnal changes.

Outcome measures

The primary outcome measures included changes from baseline in anterior segment parameters, such as front and back keratometry, central corneal thickness (CCT) at the pupil center, pupillary diameter (PD), anterior chamber angle (ACA), anterior chamber depth (ACD), and anterior chamber volume (ACV). These parameters were assessed using the Pentacam HR rotating Scheimpflug camera before and 30 minutes after tropicamide administration under standard dim light conditions.

Statistical analysis

Statistical analyses were conducted using the IBM SPSS Statistics for Windows, Version 25 (Released 2017; IBM Corp., Armonk, New York) software. Nominal and ordinal variables were analyzed using chi-square and Fisher's exact test. Changes in anterior segment parameters after cycloplegia were evaluated using Wilcoxon signed-rank test or paired-samples t-test, while parameters between groups were compared using Mann-Whitney U or independent-samples t-tests. Statistical significance was defined as P-values less than 0.05.

## Results

Following the exclusion criteria, out of the initial 144 participants assessed for eligibility, 98 eyes of 98 patients were deemed eligible and enrolled in the study. The participants were randomly allocated, with 49 patients receiving 0.5% tropicamide and 49 patients receiving 1% tropicamide, and were analyzed for primary outcome measures. The mean age of patients in the 0.5% tropicamide group was 56.22 ± 8.91 years (range: 29-72), while in the 1% tropicamide group, it was 53.37 ± 12.06 years (range: 23-76), with no significant difference observed (P = 0.19). Table 1 presents the demographic and clinical characteristics of the participants, showing no statistically significant differences in sex, duration of diabetes, or severity of diabetic retinopathy between the groups (P > 0.05).

**Table 1:** The demographic and clinical characteristics of the participants. *Chi-square test, **Fisher’s exact test. NPDR: nonproliferative diabetic retinopathy; CSME: clinically significant macular edema.

Variables	Tropicamide 0.5%	Tropicamide 1%	P-value
Sex			0.31*
Male	21	26	
Female	28	23	
Eye laterality			0.84*
Right	27	26	
Left	22	23	
Type of diabetes			0.36**
Type 1	1	4	
Type 2	48	45	
Diabetes duration (years since diagnosis)			0.37*
≤ 10	37	33	
˃ 10	12	16	
Diabetic retinopathy			0.96*
None	29	30	
Mild NPDR	9	8	
Moderate NPDR	7	8	
Severe NPDR	4	3	
CSME	0	1	1.00**
Insulin dependence	12	11	0.81*

Tables 2, 3 display the pre- and post-dilation values of front keratometry, back keratometry, CCT, and PD. Pre- and post-dilation front and back keratometry did not show significant differences within both groups (P > 0.05).

**Table 2:** The mean of corneal parameters and pupillary diameter measured by Pentacam before and after tropicamide 0.5% administration. *Paired-samples t-test. The mean values are presented as mean ± standard deviation (95% confidence interval (CI)). K1: flat keratometry; K2: steep keratometry; Km: mean keratometry; D: diopters; CCT: central corneal thickness; PD: pupillary diameter.

Variables	Pre-dilation	Post-dilation	P-value*
Front K1 (D)	43.97 ± 1.30 (43.60, 44.35)	43.93 ± 1.29 (43.56, 44.31)	0.12
Front K2 (D)	44.73 ± 1.28 (44.36, 45.10)	44.71 ± 1.28 (44.34, 45.08)	0.49
Front Km (D)	44.35 ± 1.24 (43.99, 44.71)	44.32 ± 1.24 (43.96, 44.68)	0.30
Back K1 (D)	-6.27 ± 0.23 (-6.33, -6.20)	-6.25 ± 0.22 (-6.32, -6.30)	0.20
Back K2 (D)	-6.55 ± 0.25 (-6.62, -6.47)	-6.55 ± 0.27 (-6.63, -6.48)	0.40
Back Km (D)	-6.41 ± 0.23 (-6.47, 6.34)	-6.39 ± 0.23 (-6.46, -6.33)	0.13
CCT (μm)	557.92 ± 34.96 (547.88, 567.96)	551.82 ± 32.55 (542.47, 561.17)	< 0.001
PD (mm)	2.69 ± 0.54 (2.54, 2.84)	5.68 ± 0.77 (5.45, 5.90)	< 0.001

**Table 3:** The mean of corneal parameters and pupillary diameter measured by Pentacam before and after tropicamide 1% administration. *Paired-samples t-test. The mean values are presented as mean ± standard deviation (95% confidence interval (CI)). K1: flat keratometry; K2: steep keratometry; Km: mean keratometry; D: diopters; CCT: central corneal thickness; PD: pupillary diameter.

Variables	Pre-dilation	Post-dilation	P-value*
Front K1 (D)	43.64 ± 1.48 (43.21, 44.06)	43.59 ± 1.51 (43.15, 44.02)	0.12
Front K2 (D)	44.43 ± 1.53 (43.99, 44.87)	44.40 ± 1.55 (43.95, 44.84)	0.25
Front Km (D)	44.03 ± 1.48 (43.60, 44.46)	43.99 ± 1.51 (43.56, 44.42)	0.18
Back K1 (D)	-6.23 ± 0.26 (-6.30, -6.15)	-6.22 ± 0.25 (-6.30, -6.15)	0.77
Back K2 (D)	-6.50 ± 0.28 (-6.58, -6.41)	-6.51 ± 0.28 (-6.59, -6.43)	0.051
Back Km (D)	-6.36 ± 0.26 (-6.44, -6.29)	-6.36 ± 0.25 (-6.43, -6.29)	0.57
CCT (μm)	547.55 ± 35.80 (537.26, 557.84)	541.16 ± 33.22 (531.62, 550.70)	< 0.001
PD (mm)	2.85 ± 0.56 (2.69, 3.01)	5.96 ± 0.66 (5.77, 6.14)	< 0.001

However, the study showed a statistically significant decrease in the mean CCT following cycloplegia in both groups (P < 0.001). The mean changes in front and back keratometry, CCT, and PD (Table 4) did not differ significantly between the groups (P > 0.05).

**Table 4:** The comparison of mean changes in corneal parameters and pupillary diameter measured by Pentacam after tropicamide administration between the two groups. *Independent-samples t-test. The mean values are presented as mean ± standard deviation (95% confidence interval (CI)). K1: flat keratometry; K2: steep keratometry; Km: mean keratometry; D: diopters; CCT: central corneal thickness; PD: pupillary diameter.

Variables	Tropicamide 0.5%	Tropicamide 1%	P-value*
Front K1 (D)	-0.04 ± 0.18 (-0.09, 0.01)	-0.05 ± 0.23 (-0.12, 0.01)	0.80
Front K2 (D)	-0.02 ± 0.22 (-0.09, 0.04)	-0.04 ± 0.23 (-0.11, 0.03)	0.72
Front Km (D)	-0.03 ± 0.19 (-0.08, 0.03)	-0.04 ± 0.21 (-0.10, 0.02)	0.76
Back K1 (D)	0.01 ± 0.07 (-0.01, 0.03)	0.002 ± 0.05 (-0.01, 0.02)	0.39
Back K2 (D)	-0.01 ± 0.07 (-0.03, 0.01)	-0.01 ± 0.05 (-0.03, 0.00)	0.61
Back Km (D)	0.01 ± 0.05 (-0.003, 0.02)	0.004 ± 0.05 (-0.01, 0.02)	0.53
CCT (μm)	-6.10 ± 8.06 (-8.42, -3.79)	-6.39 ± 9.97 (-9.25, 3.52)	0.88
PD (mm)	2.99 ± 0.62 (2.81, 3.16)	3.11 ± 0.55 (2.95, 3.26)	0.31

Table 5 presents the mean ± SD values of ACA, ACD, ACV, and horizontal WTW measured by Pentacam. Both 0.5% and 1% tropicamide resulted in a significant decrease in ACA (P < 0.001, P = 0.001, respectively) and a significant increase in WTW (P < 0.001, P = 0.03, respectively), ACD (P < 0.001 for both), and ACV measurements (P < 0.001 for both). However, the mean changes in ACA, ACD, ACV, and WTW were not significantly different between the two groups (P > 0.05).

**Table 5:** The mean values and changes of anterior chamber parameters and white-to-white distance measured by Pentacam before and after tropicamide administration. *Wilcoxon signed-rank test, **Mann-Whitney U test. The mean values are presented as mean ± standard deviation (95% confidence interval (CI)). ACD: anterior chamber depth; ACA: anterior chamber angle; ACV: anterior chamber volume; WTW: white-to-white distance.

Variables	Tropicamide 0.5%	Tropicamide 1%	P-value**
ACA (degree)			
Pre-dilation	30.31 ± 4.72 (28.95, 31.66)	32.70 ± 6.45 (30.84, 34.55)	0.10
Post-dilation	26.80 ± 10.11 (23.90, 29.71)	29.40 ±10.00 (26.52, 32.26)	0.10
The change	-3.50 ± 10.65 (-6.56, -0.44)	-3.30 ± 6.87 (-5.28, -1.32)	0.31
P-value*	< 0.001	0.001	
ACD (mm)			
Pre-dilation	2.51 ± 0.33 (2.41, 2.60)	2.61 ± 0.34 (2.51, 2.71)	0.23
Post-dilation	2.60 ± 0.34 (2.50, 2.70)	2.71 ± 0.35 (2.61, 2.81)	0.24
The change	0.10 ± 0.05 (0.08, 0.11)	0.10 ± 0.05 (0.08, 0.11)	0.10
P-value*	< 0.001	< 0.001	
ACV (mm^3^)			
Pre-dilation	118.20 ± 30.24 (109.52, 126.89)	132.77 ± 37.15 (122.10, 143.44)	0.07
Post-dilation	134.90 ± 28.62 (126.67, 143.12)	150.28 ± 36.32 (139.85, 160.72)	0.04
The change	16.69 ± 9.56 (13.95, 19.44)	17.51 ± 9.26 (14.85, 20.17)	0.89
P-value*	< 0.001	< 0.001	
WTW (mm)			
Pre-dilation	11.49 ± 0.42 (11.37, 11.61)	11.62 ± 0.47 (11.49, 11.76)	0.18
Post-dilation	11.55 ± 0.39 (11.43, 11.66)	11.66 ± 0.51 (11.51, 11.81)	0.17
The change	0.06 ± 0.14 (0.02, 0.10)	0.03 ± 0.30 (-0.05, 0.12)	0.054
P-value*	< 0.001	0.03	

## Discussion

The current study aimed to investigate the effects of two concentrations of tropicamide on anterior segment parameters using Pentacam in diabetic patients. Our discussion focused on several key aspects, including CCT, keratometry, horizontal WTW, ACD, ACV, and ACA.

While it is theorized that the paralysis of the ciliary muscle induced by the cycloplegic drug reduces the force exerted on the scleral spur, leading to corneal flattening [10], our study found no significant changes in keratometry following tropicamide administration, an essential evaluation before keratorefractive surgeries. Although changes are not clinically or statistically significant, it is speculated that diabetic autonomic neuropathy may weaken the ciliary muscle and diminish its response to cycloplegics, reducing the difference in forces exerted on the scleral spur before and after cycloplegia. This could result in nonsubstantial alterations in corneal curvature, warranting further investigation for elucidation.

We did observe a decrease in CCT post-tropicamide after cycloplegics, consistent with previous findings [11]. Studies investigating the effects of cycloplegics on CCT have yielded conflicting results, with some reporting increases attributed to various factors such as aging, medication type, and tear film changes. In contrast, others, including our study, have observed decreases or no significant changes [5,11-15]. Despite conflicting results in the literature, relying on pre-dilation CCT values remains a rational approach for diabetic patients undergoing corneal ablation procedures.

Moreover, a significant widening of the horizontal WTW was observed following tropicamide administration, which is in line with previous findings [5,16]. This alteration could have implications for angle-supported and phakic intraocular lens (IOL) implantation, underscoring the importance of considering such changes during surgical planning. Additionally, horizontal WTW and keratometry serve as reference values for determining the appropriate base curve and diameter of contact lenses to be worn.

Moving on to ACD and ACV, significant increases were observed post-tropicamide administration, aligning with prior literature attributing such increments to decreased lens thickness and iris displacement [5,16]. However, it is important to note that Pentacam may show discrepancies in ACD and ACV values compared to other imaging modalities. Although these differences may not affect IOL power calculation, their relevance in estimating the safety of phakic IOL vaults requires further investigation [4].

Regarding ACA, a significant narrowing of the angle was observed post-tropicamide administration [5,16], contrary to some previous findings reporting angle widening [17]. This discrepancy underscores the need for additional research to explore variations in ACA measurements among different anterior segment imaging devices, particularly in diabetic patients.

In addition to the discussed findings, it is crucial to acknowledge that while measurements with Pentacam were deemed precise, repeatable, and reproducible, many of the differences observed post-dilation in the current study fell within the repeatability intervals of the measurements [18,19]. This highlights the importance of considering the inherent variability in measurements when interpreting the results. Therefore, caution should be exercised in drawing conclusions solely based on small changes observed within the repeatability thresholds.

Furthermore, although it is established that different devices should not be used interchangeably [4,20], comparing values between various devices and considering a safer margin may be beneficial, particularly in candidate selection for refractive surgeries. By incorporating a margin of safety and considering potential measurement variations between devices, clinicians can enhance decision-making and minimize the risk of adverse outcomes in patients undergoing such procedures.

However, the current study has limitations, including its single-center nature and small sample size. Despite efforts to address potential confounders, such as diabetes duration, including patients with varying durations of diabetes in the study criteria might have introduced heterogeneity in the patient population, potentially affecting the generalizability of the findings. This factor should be taken into consideration when interpreting the results, as the duration of diabetes can potentially influence diabetic changes and lead to varied responses to cycloplegics in diabetic patients. Stratifying a larger sample size based on age, refractive error [21], and diabetes duration may provide a more comprehensive analysis of changes.

## Conclusions

In summary, the current data provide valuable insights into the effects of tropicamide-induced cycloplegia on anterior segment parameters in diabetic patients. While changes in CCT and WTW may not always hold clinical significance, meticulous preoperative undilated assessment remains crucial for optimizing keratorefractive surgical outcomes in this population. Moreover, although changes in ACD, ACV, and ACA may fall within clinically tolerable ranges for IOL calculation, consideration of undilated measurements in diabetic patients who are candidates for phakic IOL implantation may be warranted.
